# Revealing Natural Relationships among Arbuscular Mycorrhizal Fungi: Culture Line BEG47 Represents *Diversispora epigaea*, Not *Glomus versiforme*

**DOI:** 10.1371/journal.pone.0023333

**Published:** 2011-08-11

**Authors:** Arthur Schüßler, Manuela Krüger, Christopher Walker

**Affiliations:** 1 Department of Biology, Biocenter of the Ludwig-Maximilian-University Munich, Martinsried, Germany; 2 Royal Botanic Garden Edinburgh, Edinburgh, United Kingdom; 3 School of Earth and Environment, University of Western Australia, Western Australia, Australia; New York State Health Department and University at Albany, United States of America

## Abstract

**Background:**

Understanding the mechanisms underlying biological phenomena, such as evolutionarily conservative trait inheritance, is predicated on knowledge of the natural relationships among organisms. However, despite their enormous ecological significance, many of the ubiquitous soil inhabiting and plant symbiotic arbuscular mycorrhizal fungi (AMF, phylum *Glomeromycota*) are incorrectly classified.

**Methodology/Principal Findings:**

Here, we focused on a frequently used model AMF registered as culture BEG47. This fungus is a descendent of the ex-type culture-lineage of *Glomus epigaeum*, which in 1983 was synonymised with *Glomus versiforme.* It has since then been used as ‘*G. versiforme* BEG47’. We show by morphological comparisons, based on type material, collected 1860–61, of *G. versiforme* and on type material and living ex-type cultures of *G. epigaeum*, that these two AMF species cannot be conspecific, and by molecular phylogenetics that BEG47 is a member of the genus *Diversispora*.

**Conclusions:**

This study highlights that experimental works published during the last >25 years on an AMF named ‘*G. versiforme*’ or ‘BEG47’ refer to *D. epigaea*, a species that is actually evolutionarily separated by hundreds of millions of years from all members of the genera in the *Glomerales* and thus from most other commonly used AMF ‘laboratory strains’. Detailed redescriptions substantiate the renaming of *G. epigaeum* (BEG47) as *D. epigaea*, positioning it systematically in the order *Diversisporales*, thus enabling an evolutionary understanding of genetical, physiological, and ecological traits, relative to those of other AMF. *Diversispora epigaea* is widely cultured as a laboratory strain of AMF, whereas *G. versiforme* appears not to have been cultured nor found in the field since its original description.

## Introduction

A solid phylogeny is the basis for natural systematics and the understanding of hierarchical levels in taxonomy and functional diversity of organisms. This is particularly important for those organisms that are widely used in basic research and are commonly known as model species. Here, we clarify and rectify the systematic classification of an experimentally frequently used arbuscular mycorrhizal fungus (AMF). This fungus, catalogued as BEG47, is phylogenetically distinct from most other laboratory strains affiliated with the genus *Glomus*, but since the early 1980s has erroneously been known as *Glomus versiforme*.

Fungi forming arbuscular mycorrhiza (AM) are main drivers of most terrestrial ecosystems, living in intimate mutualistic symbiosis with the majority of vascular land plants, which they provide with water and inorganic nutrients, mainly phosphorus (P). Because most crop plants form AM, and global P deposits are on the verge of depletion, AMF can be considered indispensable for sustainable agriculture. It will thus become very important to better understand the biology and ecology of individual AMF species. The fact that they are asexual, multikaryotic, and obligately biotrophic, however, makes their study complicated and difficult. All AMF are placed in the monophyletic fungal phylum, *Glomeromycota* [1]. In the past, morphological classification often yielded taxonomic groupings that did not reflect natural relationships. Fortunately, such misclassifications are now less frequent as DNA based characterisation becomes more common.

Many AMF formerly assigned to the genus *Glomus*, based on a limited number of morphological characters, have now been shown to belong to any one clade of the four presently described orders of the *Glomeromycota*, separated by hundreds of millions of years of evolution. For example, the former *G. occultum* and its relatives were shown to belong to an ancient lineage [2] and consequently transferred to *Paraglomus* in the *Paraglomeraceae* [3], which later was assigned to a separate order, the *Paraglomerales* [1]. Likewise, *G. callosum* and *G. gerdemannii* are now placed in the genus *Ambispora* [4]–[5] (*Archaeosporales*), another basal glomeromycotan lineage. Many systematically misplaced species were thus transferred from *Glomus* to other genera, in agreement with a natural classification [6], and recently several species from the phyloclade *Glomus* Group C (GlGrC, [7]) have been transferred to the genus *Diversispora* (*Diversisporales*) [8]. Nonetheless, there are many species still called *Glomus*, which remain to be correctly placed once their phylogenetic affiliation is known.

A natural classification system is crucial for the description and understanding of phylogenetic, functional and trait diversity that influence patterns of plant and AMF community productivity. Plant phylogenetic diversity is possibly correlated with community productivity through functional diversity, and high AMF diversity has been shown to promote plant diversity and also plant community productivity [9]–[10]. Functional differences of AMF and plants must impact upon each other and order- or family-level phylogenetic relations, or both, have been shown to determine AMF community assemblies and mycorrhizal symbiotic functioning [11]. Phylogenetic affiliation may also be important for understanding functioning at the molecular level, as might, for example, be indicated by differential gene expression and pathogen resistance upon colonization by either culture DAOM197198 (as *G. intraradices*, *Glomerales*), BEG47 (as *G. versiforme*) or *Gigaspora gigantea* (*Diversisporales*) [12]. In this instance, BEG47, although named ‘*Glomus*’, is a species from the *Diversisporales* and thus more closely related to *Gigaspora* than to ‘*G. intraradices*’ DAOM197198.

As previously presented for the ‘model fungus’ in AM research, DAOM197198 [13] (now *Rhizophagus irregularis*: synonym *G. irregulare*, [8] [14]), we here present a detailed review of the phylogenetic position of BEG47, which is probably the second most often used AMF culture in basic research and molecular biological studies (e.g. [15]–[17]). The type material of both, *G. epigaeum* and *G. versiforme* (synonym *Endogone versiformis*) and the synonymisation [18] of BEG47 with *G. versiforme* were re-examined.

The species under consideration in relation to BEG47 are:

i) *Endogone versiformis*, named from combined collections (November 1860 to January 1861) [19] and deposited in the Helsingfor Botanic Garden, Helsinki (H) by W. Nylander. The species was later transferred to the genus *Glomus* as a heterotypic synonym of *G. macrocarpus* var. *macrocarpus* [20] and then recognised as not conspecific with *G. macrocarpum*, and classified as *G. versiforme* [18].

ii) *Glomus epigaeum* (described as *G. epigaeus*) [21], synonymised as a later heterotypic synonym of *G*. *versiforme* [18]. The species was described from a pot culture at Oregon State University, numerous subcultures of which have been extensively used for research, as *G. epigaeus* [22], as *G. epigaeum* [23] and, most commonly, as *G. versiforme* (e.g., [15]–[17] [24]–[25]). The culture-line used in basic research, which includes BEG47, stems from the original multi-spore culture from which *G. epigaeum* was described in 1979 [21].

This study aimed at substantiating the phylotaxonomic affiliation of BEG47 and clarifying its phylogenetic relationship within the *Diversisporaceae*. We also included some other species recently transferred from *Glomus* to *Diversispora* and *Redeckera* [8] and considered, in addition, the environmental sequences of *Diversisporaceae* from public databases to analyse the global distribution of species from the *Diversisporaceae*. These data will also facilitate future molecular ecological, evolutionary and taxonomic studies, as they are currently implemented in a third party annotated, web-accessible database [26] for reliable analyses based on well-annotated fungal sequences.

## Results

The culture-line represented by BEG47, which was already known to be phylogenetically distinct from most other species in *Glomus* [27]–[28], produces both pale (e.g. W5167/Att475-45) and darkly coloured (e.g. W5165/Att475-45) spores. The pale spores (which are considerably larger than the size range given for *E. versiformis* [ = *G. versiforme*] and may darken with age) are characterized by the same rDNA sequence types as the darker ones and thus are doubtless conspecific.

### Molecular phylogeny of *Diversispora epigaea* BEG47 and *Diversisporaceae*

**Figure 1:**
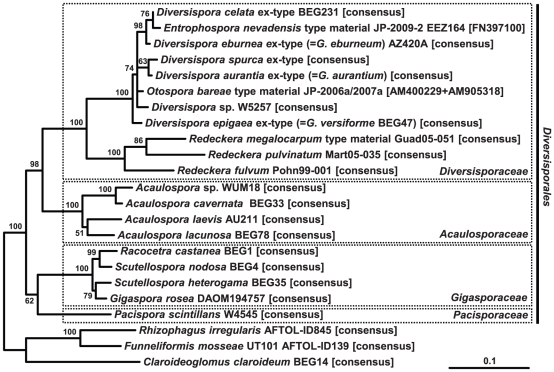
Phylogenetic tree of *Diversisporales* computed from the core dataset of nuclear SSU-ITS-LSU rDNA sequences. RAxML maximum likelihood analysis with bootstrap support shown at the branches; topologies with support below 50% were collapsed to polytomies. The most recent synonyms for species in *Diversispora* are given in brackets. The published ‘*Entrophospora nevadensis*‘ sequence (SSU rDNA) is short and does not allow species resolution, but clusters with high support within the *Diversispora celata* - *D*. *eburnea* clade. The two short, concatenated ‘*Otospora bareae*‘ sequences (SSU rDNA) also cluster within the genus *Diversispora*. The genus *Redeckera* comprises the species formerly published as *Glomus fulvum*, *G. megalocarpum* and *G. pulvinatum*. The tree is rooted with three representative sequences of the sister order *Glomerales*. The scale bar indicates proportional substitutions per site.

To study the phylogenetic relationships in greater detail, a core sequence dataset was analysed consisting of all *Diversisporaceae* sequences available, except environmental sequences lacking species assignment. The internal transcribed spacer (ITS) and partial large subunit (LSU) rDNA regions of the generic type species, *D. spurca*, were also characterised. The phylogenetic analysis (Figure 1) clearly shows that *G. epigaea* ( = *G. versiforme* BEG47), *G. aurantium*, *G. eburneum*, and *G. trimurales* all belong to *Diversispora*, in the *Diversisporaceae*, in agreement with the recent major taxonomic revision of *Glomeromycota* [8]. *Redeckera* is well separated from *Diversispora*, justifying its generic status as already suggested by Redecker and colleagues [29].

**Figure 2:**
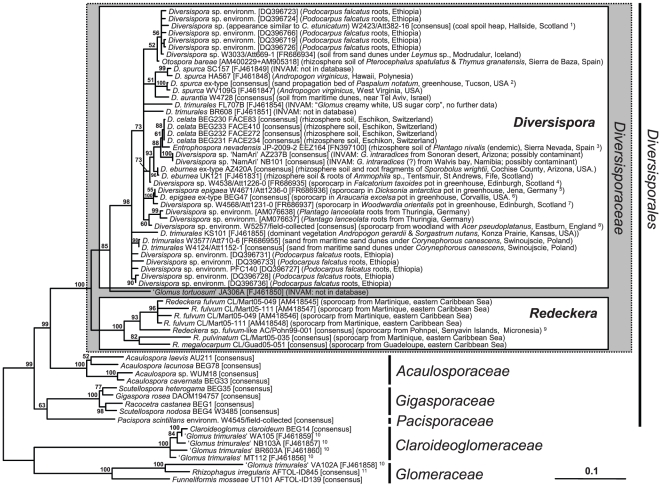
Phylogenetic tree of *Diversisporaceae* computed from the extended dataset, including environmental nuclear rDNA sequences. RAxML maximum likelihood analysis with bootstrap support shown at the branches; topologies with support below 50% were collapsed to polytomies. The tree is rooted with representatives of the *Glomerales*. The scale bar indicates proportional substitutions per site. Except for very short environmental SSU rDNA sequences that distorted the tree topology, all *Diversisporaceae* sequences which were available from the public databases were used and have the following origins: ^1^ the specimen from which this sequence was derived has *Claroideoglomus etunicatum*-like spore morphology; soil from a re-vegetated coal spoil heap, beneath *Salix* sp. and associated weeds, which included *Plantago major*, *P. lanceolata*, *Fragaria vesca* and various grasses; ^2^ Fazio's Greenhouse, from M. Pfeiffer's pot culture no. 157, Building 42-2R, University of Arizona; ^3^ other plants reported at the soil sampling location were *Alchemilla fontqueri* and *Senecio elodes* (both endemic) and *Sorbus* hybrid (non-endemic); ^4^ fungus with an appearance similar to a ‘large-spored *D. epigaea*’, from a temperate greenhouse of Royal Botanical Garden Edinburgh, Plant No. 842581 H; ^5^ immature spores; from fern house of Botanical Garden Jena (the plant was transferred to Jena from the botanical garden of the Wilhelma, Stuttgart, Germany); ^7^ *Diversispora epigaea*-like spores; temperate greenhouse of Royal Botanical Garden Edinburgh, the pot also contained an *Oxalis* sp. as a weed; ^6^ tropical greenhouse at the USDA-ARS horticultural research station; ^8^ sporocarp from litter layer of semi natural woodland, with associated understory, including an *Allium* sp.; ^9^ this sequence most likely represents a species distinct from *Redeckera fulvum*, therefore it is annotated here as ‘*R. fulvum*-like’; ^10^ sequences annotated as ‘*D. trimurales*’, from the same submission as the three sequences (FJ461851,54,55) that cluster in *Diversispora*, but clearly falling in distinct families; ^11^ culture published as GINCO4695rac-11G2 from the AFTOL project, but lacking further information.

The extended dataset contained environmental sequences carrying sufficient phylogenetic information for analysis below genus level (Figure 2), although the sequences that vary greatly in length did not always overlap in the multiple alignment. From non-monophyletic clustering of such non- or partly-overlapping sequences it is impossible to prove whether or not they are of conspecific origin. A couple of short environmental database SSU rDNA sequences were omitted from the analysis shown in Figure 2 because they lowered phylogenetic resolution and disturbed tree-topologies. They all clustered within *Diversispora* at the generic level (Figure S1), except one environmental sequence (DQ357079) from *Ammophila arenaria* rhizosphere soil from Portugal, which clusters basally in the *Diversisporaceae*. The geographical annotations of sequences falling within the phylogenetic lineage of *Diversispora* indicate a panglobal distribution of the genus, through Europe, Africa, Asia, Hawaii, the Middle East, North America and Central America (Figure 2; Figure S1).

### Morphology of the spores in the type material of *Endogone versiformis* (*G. versiforme*)

**Figure 3:**
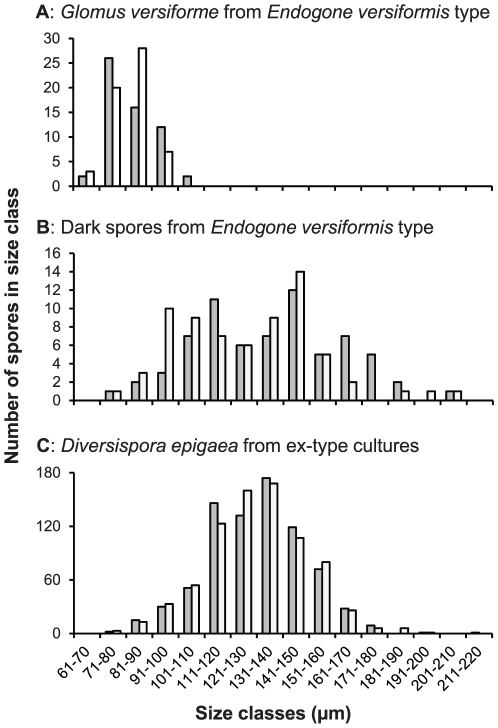
Dimensions of spores from *Glomus versiforme* type collection and *of Diversispora epigaea* (grey: lengths and white: width). **A**. Spores of the lectotype of *Glomus versiforme* (W4551) prepared from the *Endogone versiformis* type material. **B**. Large spore type (W4550) of an unknown species in the *E. versiformis* type material. **C**. *Diversispora epigaea* BEG47 (combined measurements of specimens from 49 voucher collections sampled from among 29 ex-type sub-cultures).

**Figure 4:**
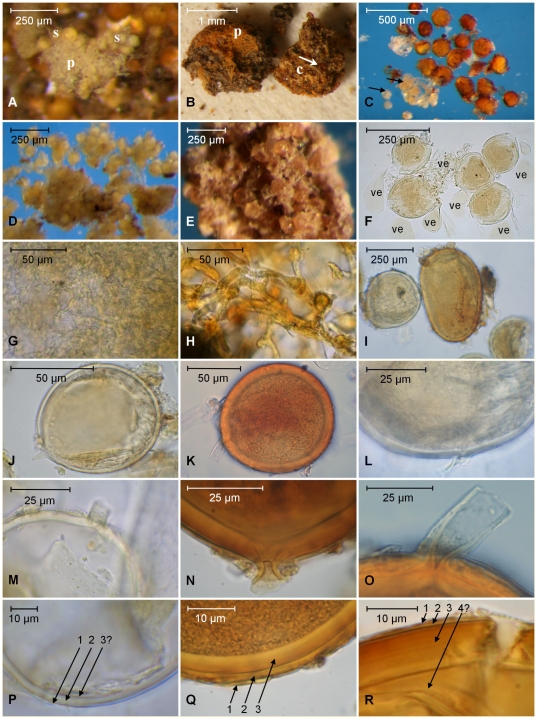
Photomicrographs of specimens from the holotype collection of *Glomus versiforme* (basionym *Endogone versiformis*). Pale spores (*G. versiforme*) of W4551, dark spores (undetermined *Glomus* sp.) of W4550. **A**. Sporocarp portion of *G. versiforme* showing pale spores (s) and a felted, pale-coloured peridium (p). Larger, dark coloured spores of an unknown *Glomus* sp. can be seen out of focus in the background. **B**. Part of a sporocarp of *Glomus* sp. showing the pigmented peridial (p) and contextual (c) hyphae and embedded spores (arrows). **C**. The two different spore morphs in water (*G. versiforme* indicated by arrows), illustrating the difference in spore size and colour. **D**. The pale-coloured spores of *G. versiforme* showing clustered spores from a sporocarp. **E**. Sporocarp portion of the dark spored unknown *Glomus* sp. **F**. Five clustered spores of *G. versiforme* from a sporocarp with accompanying vesicles (ve). **G**. Peridial hyphae of *G. versiforme* showing size and colour. **H**. Peridial hyphae of the dark spored *Glomus* sp. **I**. Spores of *G. versiforme* (left) and of the dark spored *Glomus* sp. (centre), allowing comparison of size, shape and pigmentation. **J**. Thin-walled pale-coloured spore of *G. versiforme*. **K**. A thick-walled darkly coloured spore of *Glomus* sp. **L** and **M**. Subtending hyphae of *G. versiforme*. Most specimens are sessile because of breakage of the very thin subtending hyphal wall at the spore base. **N** and **O**. Subtending hyphae of the dark spored *Glomus* sp., broken close to the spore base and occluded by an amorphous plug in the bowl-shaped lumen (N) or persistent and occluded by spore wall thickening (O). **P**. Wall detail of a spore of *G. versiforme* showing two components in the structural spore wall (1, 2) and a questionable third component internally (3?). **Q** and **R**. Wall detail of a spore of the dark spored *Glomus* sp. showing three components in the structural spore wall (Q), and a possible fourth (4?) separate component (R) internally.

The herbarium packet was annotated ‘Type of *Endogone versiforme* Karst. DET: S. M. BERCH DATE: AUG 25, 1983’. The sample was accompanied by a note with sketches in ink, dated ‘nov.1860’. The note is expanded with additional drawings and further annotation in pencil, indicating that it was originally in the hand of W. Nylander; however, the additional drawings are unsigned and it could not be established when or by whom they were made. The original notes on the type material, together with the translation into English of the Latin descriptions and annotations, are shown in Figure S2 and the spore dimensions are given in Figure 3. The type consisted of two small packets, each containing a very small quantity of dried substrate incorporating a few very small fragments of sporocarps (Figure S3). No prepared microscope slides or other preserved material were included. Examination of the holotype material of *G. versiforme* (Figure 4) shows that it contained two rather distinctive kinds of spores (Figure 4A–C,I), found either individually in the substrate or as fragments of sporocarps (Figure 4A–E). One morph consists of small, pale spores (Figure 4D,F) with relatively thin walls (Figure 4J). The second morph (Figure 4K) has large, thick-walled darkly coloured spores. Both morphs are directly compared in Figure 4C and Figure 4I. The type was fractionated but it is difficult to determine if the individual spores result from disintegration of the sporocarps during almost one-and-a-half centuries of storage and handling, or if they actually were produced ectocarpically in the substrate. Nevertheless, for both morphs, spores in the sporocarps and substrate are morphologically identical.

Pale coloured spores form epigeously in sporocarps that are up to 1 cm wide (information from the protologue), though only minute fragments remain in the type collection. The sporocarp peridium has a whitish, matted appearance and consists of tightly tangled thin-walled (<1 µm thick) somewhat squamous aseptate hyphae, 3-6 µm in diameter (Figure 4G). The glebal hyphae appear tangled and are colourless, up to 15 µm wide, with very thin (<1 µm) walls.

The spores (Figure 4D,F,J) are very pale in colour (Methuen 3A3, yellow) and translucent. For 27 of 85 measured spores, it was impossible to determine the point of detachment from the subtending hypha (spore origin) and thus also to determine their lengths and widths. The dimensions of these, by simply taking the longest and shortest dimensions, were 70–104×64–91 (mean 85×77) µm. There is little variation in spore shape, and no spore was noted that exceeded the broadly ellipsoid category, defined by a maximum ratio of length to width of 1∶1.3 [30]. Of the remaining 58 spores that could be measured conventionally, 16 were broader than long. Their dimensions were 64–109×64–99 (mean, 83×82) µm. Spore shape varied little; 26 were globose, 29 subglobose, and three broadly ellipsoidal. No truly ellipsoidal (elongate, see [30]) spores were found. The structural spore wall most probably consists of two colourless components in a single group (Figure 4P). Component 1 is persistent and found on all specimens. It is up to 1 µm thick and tightly adherent to component 2 which is 2–5 µm thick. In some specimens, there appears to be a third component, <1 µm thick, but this might be an artefact caused by congealing of spore contents in these very old dried specimens. Most spores were completely detached from their subtending hypha. However, where the subtending hypha could be seen (Figure 4L,M) it was very short (no more than a few µm, but rarely up to 15 µm long), with a very thin (≤1 µm) wall, up to 7 µm wide distally, and usually tapered sharply proximally to a width of ∼1 µm. Hyphal attachments appear to be occluded by fusing of the spore wall internally.

Redescription of **Glomus versiforme** (P. Karst.) S. M. Berch (MycoBank **MB106567**) ≡ *Endogone versiformis* P. Karst (MycoBank **MB372848**) (Figure 4A,D,F,G,J,L,M,P).

Sporocarps of indeterminate size and irregular shape, with a pale, felty peridium; protruding through, or on the surface of substrate. Spores globose to subglobose to broadly ellipsoid, 64–109×64–99 (mean, 83×82) µm, with a subtending hypha, often truncated proximally and difficult or impossible to locate. Sealed by a septum-like structure apparently formed from the inner layers of the main structural wall component. Wall structure of an outer, unit wall component (up to 1 µm thick) adherent to an inner, laminated main structural component, 2–5 µm thick, both being continuous with the wall of the subtending hypha, and thus presumably of the sporogenous mycelium. Spores in sporocarps accompanied by thin-walled (<1 µm), balloon-shaped vesicles, 41–92×61–196 µm.

Mycorrhizal status unknown, but by analogy with other members of the *Glomeromycota*, and considering that the specimens came from potted plants in a greenhouse, it is likely that *G. versiforme* forms AM.

Specimens examined: **Finland**, Nylandia, Helsingfors (Helsinki). Spores and fragments of sporocarps from the potting substrate of *Cercocarpus ledifolia* grown in a cold glasshouse, ‘23. XI. 1860 – I. 1861’ [*sic*], leg. W. Nylander (Mus. Bot. Univ., Helsinki 3936 p.p. H – Lectotype [Voucher W4551 (H, isolectotype E)]).

Dark coloured spores form in sporocarps, embedded in coarse, reddish yellow glebal hyphae, and ectocarpically in the substrate (Figure 4B). Because the type sample is fragmented, it is impossible to determine the original size of the sporocarps. The spores are abundant in the substrate as individual spores and also found embedded in substrate aggregates. Therefore it appears that they can be formed ectocarpically and hypogeously. The peridium is reddish yellow (Methuen 4A6) in colour and has a woolly appearance, consisting of angular, thin-walled anastomosing coenocytic mycelium ∼3–18 µm diameter (Figure 4H). The spores (Figure 4C,E,K) are coloured variably in shades of orange to brown (Methuen 5D8–5D8), and are opaque due to their thick coloured wall (Figure 4K). Of the 121 measured spores, for 52 it was impossible to determine the location of the attachment to the subtending hypha, and thus impossible to distinguish lengths from breadth. By simply taking longest and shortest dimensions, the resulting size range was 73–208×73–208 µm (mean 137×128 µm). There is considerable variation in spore shape, and many spores exceeded the broadly ellipsoid category and were ellipsoidal. Of the remaining 69 spores, 15 were broader than long and 47 were longer than broad. The shape of the spores varied considerably. Seven were globose, 32 subglobose, 20 were broadly ellipsoidal, and 10 were ellipsoidal (elongate).

The spore wall consists of three, possibly four, components (Figure 4Q,R). Component 1 at first is thin, ∼1 µm thick. It appears to expand to become as much as 4 µm thick, and eventually to disintegrate and disappear, and thus can be classified as evanescent as defined by Walker [31]. It tightly adheres to component 2, a unit component that varies in thickness from 1–5 µm. Wall component 3 is 5–12 µm thick and very finely laminated, though the laminations often are difficult to distinguish. In many specimens, there seems to be a fourth thin flexible inner component 4 (Figure 4R), though on others it was not detectable (Figure 4Q). It is not clear if this is an artefact of specimen preparation such as a loose lamina of component 3, but it is evident in both glycerol and PVLG-based preparations. The wall thins at the spore base to produce a bowl-shaped lumen 3–10 µm diameter internally, tapering to ∼1 µm externally where the subtending hypha is attached (Figure 4N,O). The majority of spores have their subtending hypha detached close to the spore base. When it is retained, it is very difficult to see because it often is extremely thin-walled (normally <1 µm). It can be up to 37 µm long and as much as 15 µm wide distally, tapering to become constricted proximally to about 1 µm in diameter, where it usually becomes detached. On a few specimens, the subtending hypha is thickened to ∼2 µm proximally (Figure 4O) and sometimes it appears to be occluded by a plug of amorphous material.

### Morphology of *Glomus epigaeum* from the holotype and ex-type culture-lines, including BEG47

**Figure 5:**
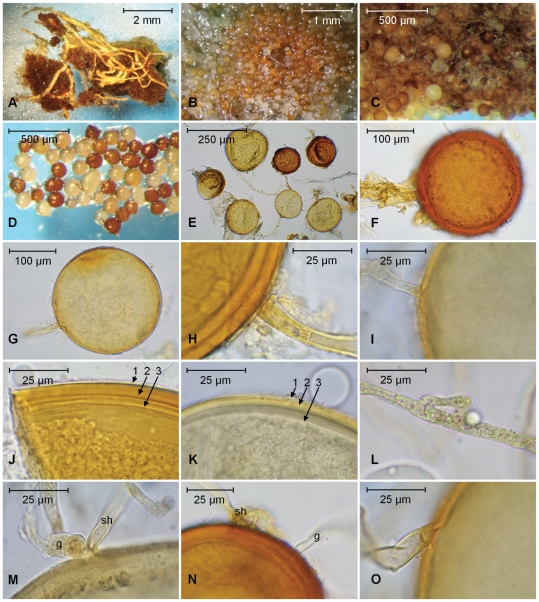
Photomicrographs of specimens from *Diversispora epigaea* ex-type pot cultures (including culture line BEG47). Dark spores of W5165, pale spores of W5167 except Figure 5L, which is from W4565. **A**. Spore cluster, formed on roots near the surface of a pot. **B**. View of a spore cluster showing the undifferentiated aggregation of pale coloured and orange spores. **C**. Spore mass, showing pale and dark spores. **D**. Spores photographed in water, uncovered on a glass microscope slide. **E**. Spores of both colours, showing variation in size, shape and pigmentation. **F**. A thick-walled pigmented spore of the dark morph. **G**. Thin-walled, immature pale-coloured spore. **H** and **I**. Subtending hyphae of dark (H) and pale (I) spores showing occlusion by spore wall thickening and a distal septum in the dark morph. Note the difference in wall thickness. **J** and **K**. Wall structure of dark (J) and pale (K), spores showing thin outer (1), thick laminated (2), and thin inner (3) components. **L**. Hyphal bridging, also known as wound healing, in the somatic mycelium. **M**. Spore germination (g) at the base of the subtending hypha (sh). **N**. Germination directly through the wall. **O**. A septum occluding the hyphal attachment of a thin-walled spore of the pale morph close to the spore base.

The spores are produced in dense masses, lacking a peridium (Figure 5A–C) and with or without varying amounts of brownish contextual hyphae, or singly (Figure 5D), or in loose clusters in the substrate. The spore masses (referred to in the protologue as ‘sporocarps’) were originally recorded as being 2–8×3–15 mm [21], but they are very variable in size and shape. The colour of the spores is variable (Figure 5B–G). They are colourless at first, soon becoming pale yellow, gradually becoming orange at maturity to dark reddish brown (Methuen 8E8) when moribund. The spore wall components do not react to Melzer's reagent, although the pale spores may become overall slightly yellow.

Seven-hundred and eighty spores were measured from among 29 ex-type cultures (Table S1; Figure 3C); 346 were broader than long, 158 were equal in length and width, and 276 were longer than broad. Spore shape was not very variable, 497 spores being globose, 212 subglobose, 56 broadly ellipsoidal, and only 15 ellipsoidal. Some of these spores were ovoid (8) or obovoid (28), two were flattened somewhat on one side, six were pyriform, and two were subtriangular. The spore dimensions were 78–213×78–192 µm (mean = 131×131 µm, n = 780). The protologue gives spore measurements for the epigeous spores as (60−)75–140(−165)×95–140 µm. In one sample, 100 dark epigeous spores and 100 pale hypogeous spores were measured separately, yielding dimensions of 82–146×85–146 µm (mean = 115×116 µm) and 85–194×96–192 µm (mean = 135×134 µm).

In some spores, the spore wall appears to have a unit outer component (Figure 5J), but on others, it breaks down in patches (Figure 5K), and thus must be considered to be evanescent. The coloured main structural component sometimes seems laminated (Figure 5H,J,K), and at other times the laminae cannot be seen by light microscopy (Figure 5I). Finally there is an innermost component (Figure 5J,K) that is often difficult to discern under the light microscope, but was described as clearly visible in transmission electron micrographs [23]. By light microscopy, the wall structure of spores in PVLG is of three components as follows: component 1 unit or more or less evanescent, colourless, up to 1 µm thick; component 2 laminated, pigmented, 1–10 µm thick depending on age; component 3 <1 µm thick, lightly pigmented, often tightly adherent to component 2 and difficult to discern, sometimes appearing flexible due to shrinkage after immersion in the mounting medium (Figure 5J,K). In a few spores the inner wall component appears to form a septum (Figure 5O). The subtending hypha is variable (Figure 5H,I,M,N,O), very narrow, not more than 10 µm at the base of the spore; straight (Figure 5I) or slightly curved (Figure 5H), or often constricted at the base (Figure 5M). Usually the subtending hyphal wall is thin (1–2 µm), tapering little in most (though not all) of the pale spores. On some mainly darkly coloured spores, the wall of the subtending hypha tapers quite sharply from up to 5 µm thick proximally (Figure 5H) to <1 µm distally where detached from the mycelium.

Germination is by emergence of a germ tube through the remnant subtending hypha or directly through the spore wall (Figure 5M,N). This species exhibits the type of self-anastomosis known as hyphal bridging (Figure 5L) or wound healing [32], also found in *D. celata* [33] and *D. spurca* [34]. This phenomenon has also been observed for members of *Ambispora, Gigaspora*, and *Scutellospora*, but differs from the formation of interhyphal anastomoses in hyphal networks of members of the *Glomeraceae* [35]–[36].

Redescription of **Diversispora epigaea** (B.A. Daniels and Trappe) C. Walker and A. Schüßler (MycoBank **MB542916**) ≡ *Glomus epigaeum* (MycoBank **MB314591**) (Figure 5).

Two spore morphs (overall size range 60–213×78–192 µm), depending upon whether formed epigeously or hypogeously. Epigeous dense spore clusters, sometimes called sporocarps, irregular, known to be 2–8×3–15 mm, but seemingly indeterminate in size and shape, formed on substrate surface: peridium lacking, sometimes with a basal hyphal mat extending around the lower sides of the spore cluster. Spores globose to subglobose to broadly ellipsoid 60–170×85–174 µm, pale cream when young, becoming dull brownish yellow to orange at maturity or, at senescence, brown. Spore wall structure of three components in two groups. Wall group 1 of an evanescent component up to 1 µm thick overlaying a laminated component up to 10 µm thick. Wall group 2 of a thin (<1 µm) flexible component. Subtending hypha variable, straight or slightly curved, up to 10 µm in diameter and often constricted proximally, to 4–6 µm in diameter; subtending hyphal wall proximally up to 5 µm thick, tapering to 1 µm distally, the continuous inner wall component appearing to form an internal septum. Hypogeous spores formed singly, or in loose clusters in the soil; rarely as single spores, bursting through the root cortex; formed on colourless mycelium; colourless at first, soon becoming orange-white to light orange; globose to subglobose or broadly ellipsoid 85–213×96–192 µm. Wall structure and subtending hypha as for epigeous spores. Neither hypogeous nor epigeous spores react in PVLG-Melzer's or pure Melzer's reagent except to become slightly yellowish (contents sometimes becoming orange). Anastomosis of the type known as hyphal bridging (wound healing) present in extraradical somatic mycelium.

Forming arbuscular mycorrhiza with numerous hosts including Araucaria excelsa [21], Asparagus officinalis, Sorghum bicolor, Allium porrum, Plantago lanceolata, Trifolium repens, Lotus japonicus and Festuca ovina (see Table S1).

Specimens examined: Spores and spore clusters from the type material and 29 other ex-type collections from cultures maintained in the USA, UK, Italy, France, Belgium, Finland and Germany (see Table S1).

## Discussion

### *Glomus versiforme ( = Endogone versiformis)*

The epithet given by Karsten [19], *versiforme,* indicates variability although in the protologue there is no mention of extreme variation or of the presence of two morphs in the type material. Obviously, only the paler morph was included in the species circumscription of W. Nylander (Figure S2), and this has been followed by Karsten [19] in his species description, which is brief, but specific. It describes the spores as globose and white, and gives spore dimensions (65–95 µm) that fit only with the smaller of the two morphs. The size range we measured for the pale-coloured spores in the type material of *G. versiforme* corresponds well with that of the protologue of that species. Both the size and appearance of these are very different from those of the larger, orange and more ovoid spores in the substrate comprising the type material. The smaller paler-coloured spores were produced in sporocarps with a pale coloured peridium with white woolly elements, specified in the protologue as a feature of *E. versiformis*. The larger, darkly-coloured spore clusters come from sporocarps with darkly coloured peridial hyphae. With the description of spore colour, size and shape [19] this confirms the opinion that the author's intention was to apply the epithet *versiformis* only to the pale spores. The notes left by W. Nylander and the pencilled annotations (Figure S2) thereon also support this view. Drawings show only globose spores with a rather thin wall, relative to the spore dimensions, unlike the more darkly pigmented spores which have relatively thick walls and received no particular attention by either authority.

### *Diversispora epigaea ( = Glomus epigaeum)*

The species defined as *G. epigaeum* by Daniels and Trappe [21] and the monospecific type material lodged at OSC required little emendation with respect to its morphology. The junction of the subtending hypha is somewhat more varied than the description implies, and the statement that the subtending hypha is ‘inserted into the spore wall’ is misleading, because it is continuous with both spore components. In addition, the weak orange reaction to Melzer's reagent is in the cytoplasm, and not in the wall. Spore colour changes considerably with spore development, from nearly colourless for young spores to light orange (hypogeous spores) or dark orange for old epigeous spores. The wall structure of the spores was difficult to assess, sometimes the main structural wall appeared laminated, and other times laminations could not be detected. Because transmission electron microscopy of *D. epigaea* spores [23] showed fine laminae as twisted microfibril layers, the light microscopically visible lamination is considered not to be artefactual. Molecular phylogenetic evidence (Figure 1, Figure S1) clearly shows that BEG47 is not a member of the genus *Glomus* but belongs to *Diversispora*.

### *Glomus versiforme ( =  E. versiformis)* is a fungal species neither cultured nor re-discovered since its original description

The size, colour and nature of the peridium of the two different kinds of sporocarps in the *E. versiformis* ( = *G. versiforme*) type collection already indicate that they are unlikely to be conspecific, as indicated by differences in colour, size, form of the subtending hypha and wall structure of the smaller pale and the larger dark-spored morphs. For the pale morph most spores are more or less globose or broader than long, whereas for the dark morph most were longer than broad (we considered this significant, because the ratio of length to width has been used as a species-specific characteristic [37] [20]). The pale sporocarps of *E. versiformis* have balloon-shaped saccules amongst their spores, a feature lacking in the larger, darkly pigmented spores, which are morphologically similar to mature spores of *D. epigaea*.

Although spore size of the dark spores in the *E. versiformis* type material is not very different from those of *D. epigaea* BEG47, there are some morphological differences. In the former, hyphal attachments are rare; 68% of spores were broader than long; and there appears to be a complete peridium although only fragments of it were preserved. In contrast, for BEG47, hyphal attachments are easily found; only 44% of spores were broader than long. They are produced in large naked masses of ectocarpic epigeous spores on the surface of the substrate. Whilst it is possible that peridial development may depend on environmental conditions, true sporocarps with peridia have never been reported from cultures of BEG47 over decades of propagation in different laboratories and with different plant hosts and substrates. This further supports the distinctiveness of *D. epigaea* and both *G. versiforme* and the accompanying dark-spored fungus.

Berch and Fortin noted [18] that spores of *G. epigaeum* were much darker and larger than the description in the protologue and concluded that the spores used for the protologue were ‘probably immature’. Based on this assumption both the small, pale spores and the large, coloured spores were incorporated within a single combined description [18]. From our microscopic examination of the type material, however, we conclude that the different spore types in the type collection of *E. versiformis* most likely represent different organisms mixed in the same herbarium packet. The use of the plural (glasshouses, plants), and the dating of the collection (23.XI.1860-I.1861) in W. Nylander's notes and P. A. Karsten's protologue indicates that the type material is composed of several collections from different glasshouses and plants and thus is most likely to be mixed. The current Botanical Code dictates that type material must come from only one collection, but no such requirement applied at the time of Karsten's description.

The Botanical Code, Articles 9.9, 9.12, requires that the spore morph selected to represent *G. versiforme* from the mixed collection must be that which most closely conforms to the original diagnosis. The pale spores, presence of a ‘white-woolly’ peridium with fine hyphae and the narrow hyphal attachments therefore preclude *G. epigaeum* ( = *D. epigaea* BEG47) as a potential synonym of *G. versiforme*. Nevertheless, given that we could not obtain glomeromycotan DNA sequences from the type material of *G. versiforme*, we cannot completely exclude the possibility that the small pale and large pigmented spores in the type collection originate from a single dimorphic species, although this seems extremely unlikely. As a consequence of this notion that the original species description of *G. versiforme* was based on more than one species, a lectotype (W4551) was designated to define precisely the species [8] and to provide an emended description, based only on the pale spores (W4551). It should be noted that the new species description of *G. versiforme* is made from a combination of the original protologue and a limited number of dead spores from a mixed collection preserved in air-dried substrate for about 150 years, during which time the spores have deteriorated. To date we have not found any other conspecific specimens, nor can we find evidence that similar spores have been collected by anybody else since the original description of the species. If a representative of *G. versiforme* were to be found, it would be advantageous to define an epitype and to resolve its phylogenetic position. Without molecular evidence, the natural systematic position of *G. versiforme* must remain uncertain but morphologically, it is not conspecific with *D. epigaea*.

### BEG47 represents *Diversispora epigaea ( = G. epigaeum)* and not *Glomus versiforme ( = E. versiformis)*

Based on the present investigation, we must conclude that BEG47 is not synonymous with *G. versiforme* in the strict sense because:

a) two distinct spore morphs from more than one collection were included in the type material of *E. versiformis* ( = *G. versiforme*), most likely from two different AMF species, whereas the species description of *E. versiformis* clearly refers only to the smaller spore morph and does not mention the *D. epigaea*-like spore morph;

b) BEG47 and other *D. epigaea* ( = *G. epigaeum*) ex-type cultures do not form spores similar to the small pale spore morph in the type collection of *E. versiformis*, which represent *G. versiforme*.

Molecular evidence presented here shows BEG47 to belong to the genus *Diversispora*, and consequently, under the rules of the Botanical Code, it has to be placed in that genus as *D. epigaea*. *Diversispora epigaea* is widely cultured and frequently used as a laboratory strain for molecular, physiological and ultrastructural research, whereas *G. versiforme* appears not to have been cultured nor found in the field since its original description.

### DNA sequence annotation in the public databases

Based on previous phylogenetic analyses [6] [33] and additional data gathered during this study, *D. aurantium*, *D. eburnea*, and *D. trimurales* were also transferred from *Glomus* to *Diversispora* [8]. Several of these sequences are still annotated as ‘*Glomus*’, in the public databases. Another database sequence ascribed to *G. tortuosum* culture accession JA306A clusters basal to *Diversispora* but has to be considered of uncertain phylogenetic affiliation. No entry with the identifier JA306 could be found in the INVAM culture collection database and the sequence was included in a sequence deposition (FJ461790-FJ461888) to Genbank that likely contains mis-annotations or contaminant sequences, as for example, those attributed to ‘*G. trimurales*’ which are derived from at least three divergent AMF lineages (Figure 2). There are many sequences in the public databases that probably are incorrectly named. This problem will soon be overcome by third party annotation using the PlutoF workbench [26], through which environmental sequences from the ITS region, such as those earlier annotated as ‘uncultured *Glomus versiforme*’ from Thuringia (AM076638, AM076637), will be accessible. Species identity of these environmental sequences is not known, but is unlikely to be conspecific with *D. epigaea* (BEG47) [33], and thus should be annotated as ‘*Diversispora* sp.’. The *Diversispora* sp. sequences annotated as ‘NamAri’ from the INVAM cultures NB101 (AF185682,90-91, AF185693-95; from Namibia) and AZ237B (AF185677-81; from Arizona) are most likely of conspecific origin and are very closely related to, or perhaps conspecific with, *D. celata*. Also the short SSU rDNA sequence FN397100 ascribed to *Entrophospora nevadensis* from Sierra Nevada, Spain, is very closely related to those of *D. celata*. For the INVAM cultures NB101 and AZ237B, we suspect that the sequences could be derived from culture contaminants, wrongly determined species, or that there was a mistake made during sequence annotation, because the cultures themselves are named as ‘*G. intraradices*’ in the INVAM database. The taxonomic assignment of the sequence for *E. nevadensis* is difficult to explain. Perhaps it has been derived from a contaminant and not from the fungus morphologically described in its protologue [38], which does not share morphological characteristics with any other species in *Diversispora*.

### Biogeography of the genera *Diversispora* and *Redeckera* (*Diversisporaceae*)

Members of the genus *Diversispora* appear to occur worldwide, with sequence-based records from Europe (England, Scotland, Spain, Switzerland, Germany, Poland, Estonia, Iceland), North America (California), Central America (Panama), Africa (Ethiopia), Asia (South Korea), Hawaii, and the Middle East (Israel). One sequence from Portugal (DQ357079) might be derived from another as yet undescribed genus in the *Diversisporaceae*. Habitats and hosts of *Diversispora* spp. are diverse and include some from natural and disturbed temporal and tropical ecosystems. So far, members of the genus *Redeckera* have been recorded from Guadeloupe (Caribbean Sea) and Micronesia, and one environmental sequence representing this genus originated from South Korea. Regarding the biogeography of the species in the *Diversisporaceae*, present data do not yet provide a distinct picture of global biogeography, and in some instances (e.g. for *Diversispora* sp. ‘NamAri’) the origin of the sequences seems questionable. Nevertheless, members of the genus *Diversispora* are widely distributed, reinforcing the notion that species of this genus are much overlooked although integral parts of many ecosystems [33] [45]. Improved molecular characterisation and in-field identification, in future will lead to better understanding of this ecologically and perhaps also economically significant group of AMF.

## Materials and Methods

### Generation of sequences and gathering of reference sequences

To study the phylogenetic relationships of BEG47 with other members of the *Diversisporaceae*, a core dataset was analysed that contained all available sequences of *Diversisporaceae*, except environmental sequences lacking species assignment. For the generic type species, *D. spurca*, the nuclear internal transcribed spacer (ITS) and large subunit (LSU) rDNA sequences were also characterised in this study.

For BEG47, DNA was extracted from single spores (see Table S2). PCR amplification of the near full length nuclear small subunit (SSU) rRNA gene was carried out with the primer pairs NS1/Geo10 and GeoA2/Geo11. Cloning, sequencing and sequence editing were carried out as described previously [6]. Some shorter fragments were amplified with different primer pairs, which are noted in the corresponding sequence database entries. The ITS region of nuclear rDNA was initially amplified with the primers SSU-Glom1 [39] and LSU-Glom1b (TCGTTTCCCTTTCAACAATTTCAC; [5]) or the reverse primer LR4+2 [13]. PCR was run with the Phusion High-Fidelity DNA polymerase with the following thermocycling program: 99°C denaturation for 2 min; 35 cycles of: 99°C for 10 s, 65°C for 30 s, 72°C for 60 s; final elongation at 72°C for 5 min. Later, the ITS region was amplified together with a part of the LSU rRNA gene as previously described [40]. The resulting SSU-ITS-LSU fragment covers ∼250 bp (3′ end) of the SSU rDNA, the complete ITS region including the 5.8S rRNA gene, and ∼800 bp (5′ end) of the LSU rDNA. After cloning and plasmid isolation, fragments were sequenced on an ABI automated capillary sequencer (Applied Biosystems, Forsters City, CA, USA). Electropherograms were proof-read, trimmed and assembled with SeqAssem and sequences manually aligned to a seed-alignment by using Align (both programs from Sequentix, Klein Raden, Germany; http://www.sequentix.de). The nucleotide basic local alignment search tool (nBLAST [41]) at NCBI was used to compare the new nucleotide sequences against entries in public databases and to identify diversisporacean public database sequences.

The core alignment comprised the near full-length SSU rRNA gene sequences from this study as well as such of the *Diversisporaceae* from public databases. These SSU rDNA sequences were condensed to one strict consensus sequence (coding any variable site as a degenerate base, according to IUPAC ambiguity code) if from the same fungal isolate or culture, or in one instance (*Redeckera fulvum*; synonym *G. fulvum*) from field-collected material. Details about how the strict consensus sequences were calculated are given in Table S2. The term ‘ex-type’ is used in a broad sense to indicate that the studied material is derived from a descendent of the type culture. Besides culture-derived sequences also environmental public database sequences of *Diversisporaceae* were included. An extended alignment was created for a second, broader phylogenetic analysis containing those additional short environmental sequences that did not completely disturb tree topology at the below genus level. A third dataset, used to compute the tree shown in Figure S1, additionally comprised all short environmental sequences available from the databases, including very short ones.

### Computation of phylogenetic trees

Phylogenetic maximum likelihood (ML) analyses were performed with the software RAxML through the CIPRES science gateway (http://www.phylo.org/portal2/) with the GTRGAMMA model for 1000-fold bootstrapping as well as for final tree construction. The analyses, with species from the *Glomerales* as outgroup, were based on 3043 sites from an alignment of 23 sequences (core dataset, Figure 1) or 3023 sites from an alignment of 86 sequences (extended dataset, Figure 2). Neighbour joining and parsimony analyses gave essentially the same results as the ML method (results not shown). Resulting trees were drawn in FigTree 1.3.1 (http://tree.bio.ed.ac.uk//) and edited with Microsoft PowerPoint 2007 and Adobe Illustrator CS3. New rDNA sequences were deposited in the EMBL database with the accession numbers AM713428, AM713432, and FR686934-FR686958.

### Morphology of spores, spore masses and sporocarps

Spores from pot culture substrate were extracted by centrifugation and sugar floatation [42] or by agitating and swirling in water and decanting through sieves with 35 or 50 µm openings. Selected spores were mounted in polyvinyl alcohol lactophenol (PVL) or polyvinyl alcohol lacto-glycerol (PVLG) with (PVLG/M) or without the addition of Melzer's reagent (4∶1 PVLG:Melzer's v/v) and observed through a compound microscope, with or without Nomarski differential interference contrast optics. Vouchers were stored as colonised, dried potting substrate containing roots and spores, or as semi-permanent microscope slides with specimens mounted in PVL, PVLG or PVLG/M. Vouchers, other than types, are deposited in the herbarium of the Royal Botanic Garden Edinburgh (E), along with an isolectotype of *G. versiforme* consisting of a prepared microscope slide in PVLG (Slide W4551-8). The terminology for defining spore shapes and the convention of giving spore dimensions as length by breadth, including ornamentation but excluding appendages, follows Hawksworth and colleagues [30]. Length was always taken as a perpendicular from the spore base (point of subtending hypha). Consequently, spores can be ‘broader than long’. Spore dimensions were measured on selected samples with a calibrated eyepiece graticule under a compound microscope and colours were matched with the Methuen Handbook of Colour [43]. Specimens were indexed by referring to pot cultures as Attempts (Att) and giving herbarium voucher specimens a number with a ‘W’ prefix [44], which from our own work always include microscope-slide preparations, but that may be any preserved material.

The culture tracking and specimen vouchering system allows the addition of cultures and vouchers from other sources. Thus in this study, we notionally numbered the original *Araucaria* plant, part of the plant collection in the tropical glasshouse at Oregon State University, as Att475-0 even though it was not a deliberate attempt to create a mycorrhizal pot culture. The subsequent pot culture, established by B. Daniels on asparagus with spores taken from Att475-0, was given the notional number Att475-1. The holotype of *Glomus epigaeum* (now *Diversispora epigaea*) came from this type culture pot. It was given the voucher number W90, and an authenticated sample from this pot culture, provided to C. Walker on 12 Apr 1979 by B. Daniels, was numbered W100.

The holotype of *Endogone versiformis* (now *Glomus versiforme*), loaned by the herbarium in Helsinki (H), consisted of two small packets of dried spore masses or fragments of spore masses in a gritty substrate. It included no prepared slides or other evidence of microscopic preparations, though there were some annotations by previous workers (Figures S2, S3). Type specimens were examined first dry, and then, as small subsamples, in a dish of water. Where the spore masses were sufficiently large, they were illuminated by reflected light and examined through a dissecting microscope. Colour determinations were made in comparison with standard charts, illuminated with the same light as the specimens through a split fibre optic light source at its full working voltage (colour temperature, ∼3100 K). Individual spores or very small spore clusters were selected with fine forceps and suspended in water for detailed examination.

For *G. epigaeum* we examined type or authenticated material and living ex-type subcultures such as BEG47. The type material (OSC39475) consisted of a herbarium packet that included a slide holder, labelled ‘TYPE *Glomus epigaeum* B. Daniels’, The slide mailer also has ‘Pot217’ (or ‘Pot2,7’) and ‘7/7/78’ hand printed on the upper right corner. There was also a small unlabelled vial about half full of lactophenol containing spores and spore masses. In addition, a plastic slide holder with two slides made by J. Spain, one with spores in lactophenol and one with spores in PVLG+lactophenol, was included. The former had dried out, and was re-constituted with acidified glycerol. There was also a slide (spores in what seems to be PVLG) made by S. M. Berch in 1983. The original lactophenol mounted slide (Trappe 5174) was missing. Three new slides were made by mounting spores and small fragments of spore masses in PVLG, and given the voucher numbers W90-2, W90-3, and W90-4. By deduction from the protologue and from personal communication with Barbara Hetrick (née Daniels), we determined that the type culture of *G. epigaeum* (now named *D. epigaea*) was established with *Asparagus officinalis* between autumn 1976 and an unknown date in 1977, with a single spore mass removed from a greenhouse pot with *Araucaria excelsa*. No further details of the culturing history and origin of the species are available. Thirty nine vouchers, collected from among 29 ex-type subcultures between 1979 and the present, are available from the herbarium of the Royal Botanic Garden Edinburgh (E) (C. Walker collection; see Table S1).

## Supporting Information

Figure S1 **Phylogenetic tree of** ***Diversisporaceae*** **with additional environmental nuclear rDNA sequences.** Owing to the short length of most environmental sequences several branches lack statistical support and phylogenetic resolution. RAxML maximum likelihood tree with bootstrap support shown at the branches; topologies with support below 50% were collapsed to polytomies. Sequences that were not included in the analysis shown in Figure 2 all cluster in the *Diversispora* clade, except one (DQ357079 from rhizosphere soil from Portugal), which clusters basally in the *Diversisporaceae*. The other short sequences not shown in Figure 2 originated from Great Britain, from colonised roots of *Agrostis capillaries* and *Trifolium repens* (annotated as ‘phylotype Glo12’, AF437656, AF437657) and from roots, probably of *Acer pseudoplatanus*, from an urban environment (indirect evidence, no definitive source given in database, AJ716004); from Estonia, from roots of *Fragaria vesca* (AM849266, AM849271F) sampled in a boreo-nemoral forest in Koeru and from roots of *Oxalis acetosella* (AM849285) and *Hepatica nobilis* (AM849295, AM849296, AM849307); from South Korea, Chungbuk, from *Panax japonicus* roots (EU332718, EU332719, EU332707); from U.S.A., California, from a grassland (EU123386, EU123387, EU123390, EU123394, EU123465, EU123391, EU123392); from Panama, Barro Colorado Island, from *Faramea occidentalis* seedling roots (AY129577).
(PDF)

Figure S2 **Information accompanying the** ***Endogone versiformis*** **type material.** Transcription of the handwritten labels and notes of W. Nylander (23 Nov 1860 – Jan 1861), and annotations included in the herbarium packet containing the holotype of *Glomus versiforme* (basionym *Endogone versiformis*), and their translation into English. Protologue of *E. versiformis* (Karsten 1884) and its translation into English.
(PDF)

Figure S3 **Type collection of** ***Endogone versiformis*** **.** Open herbarium packet of the type of *E. versiformis*, containing dried substrate from potted plants, with spores and fragments of sporocarps and a Petri dish (5 cm diameter) containing sporocarp fragments from the dried substrate.
(PDF)

Table S1 **List of studied samples of the** ***Diversispora epigaea*** **( =** ***Glomus epigaeum*** **) ex-type culture-line.** The culture that was registered as BEG47 is part of the ex-type culture-line of *D. epigaea*.
(PDF)

Table S2 **Composition of the strict consensus sequences used in the phylogenetic analyses.** In strict consensus sequences, site variations are coded by the IUPAC ambiguity code, thus retaining information of the source sequences as degenerate bases, unlike majority rule consensus sequences.
(PDF)
