# Supplementary material for: Revealing Natural Relationships among Arbuscular Mycorrhizal Fungi: Culture Line BEG47 Represents Diversispora epigaea, Not Glomus versiforme
Source: PLoS One. 2011 Aug 11;6(8):e23333. doi: 10.1371/journal.pone.0023333 (PMC3154914; doi:10.1371/journal.pone.0023333)
Supplement: Figure S1 — Phylogenetic tree of Diversisporaceae with additional environmental nuclear rDNA sequences. Owing to the short length of most environmental sequences several branches lack statistical support and phylogenetic resolution. RAxML maximum likelihood tree with bootstrap support shown at the branches; topologies with support below 50% were collapsed to polytomies. Sequences that were not included in the analysis shown in Figure 2 all cluster in the Diversispora clade, except one (DQ357079 from rhizosphere soil from Portugal), which clusters basally in the Diversisporaceae. The other short sequences not shown in Figure 2 originated from Great Britain, from colonised roots of Agrostis capillaries and Trifolium repens (annotated as ‘phylotype Glo12’, AF437656, AF437657) and from roots, probably of Acer pseudoplatanus, from an urban environment (indirect evidence, no definitive source given in database, AJ716004); from Estonia, from roots of Fragaria vesca (AM849266, AM849271F) sampled in a boreo-nemoral forest in Koeru and from roots of Oxalis acetosella (AM849285) and Hepatica nobilis (AM849295, AM849296, AM849307); from South Korea, Chungbuk, from Panax japonicus roots (EU332718, EU332719, EU332707); from U.S.A., California, from a grassland (EU123386, EU123387, EU123390, EU123394, EU123465, EU123391, EU123392); from Panama, Barro Colorado Island, from Faramea occidentalis seedling roots (AY129577). (PDF) [file pone.0023333.s001.pdf]

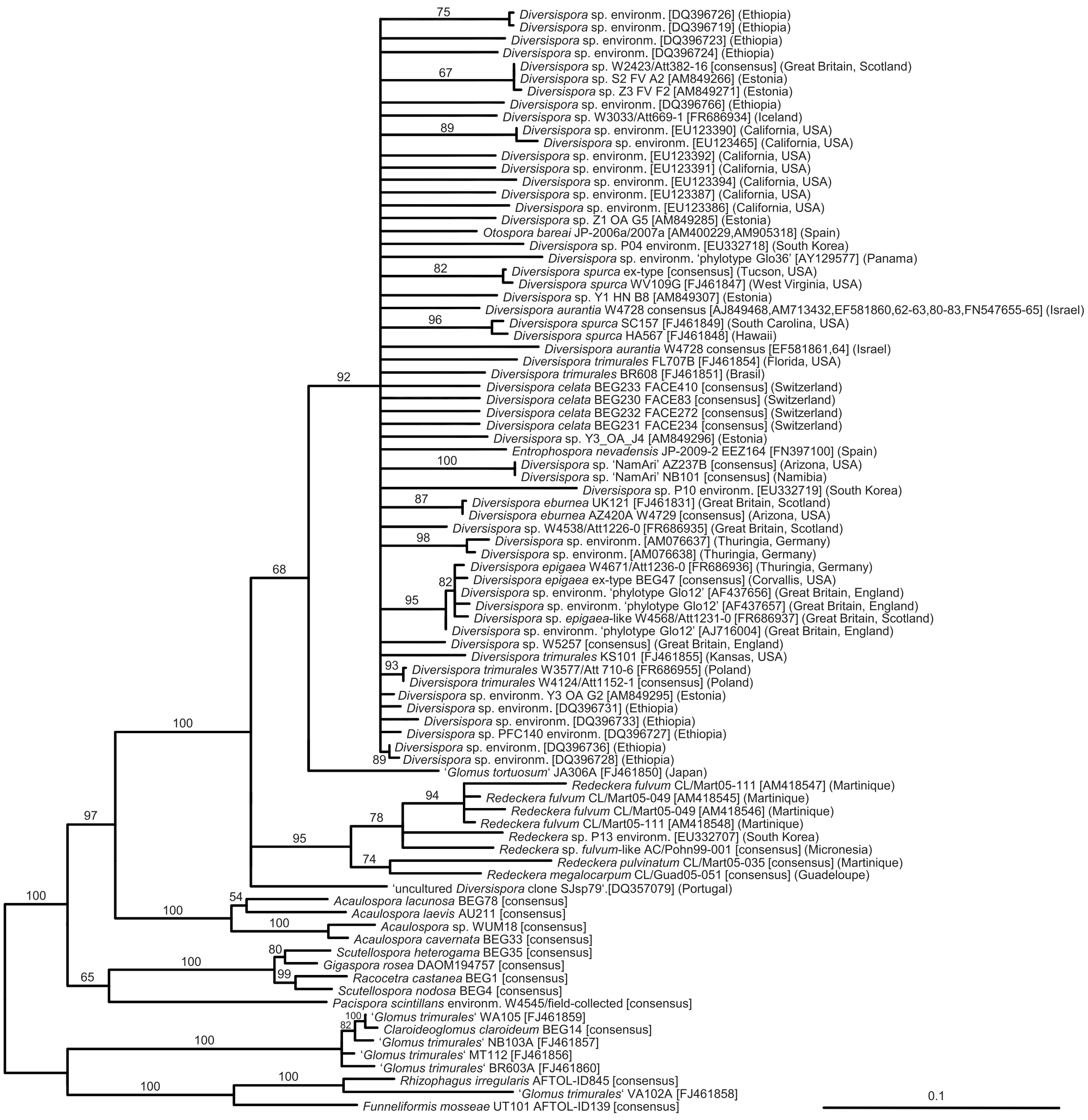

Figure S1: Phylogenetic tree of *Diversisporaceae* with additional environmental nuclear rDNA sequences.
