# Supplementary material for: Revealing Natural Relationships among Arbuscular Mycorrhizal Fungi: Culture Line BEG47 Represents Diversispora epigaea, Not Glomus versiforme
Source: PLoS One. 2011 Aug 11;6(8):e23333. doi: 10.1371/journal.pone.0023333 (PMC3154914; doi:10.1371/journal.pone.0023333)
Supplement: Figure S2 — Information accompanying the Endogone versiformis type material. Transcription of the handwritten labels and notes of W. Nylander (23 Nov 1860 – Jan 1861), and annotations included in the herbarium packet containing the holotype of Glomus versiforme (basionym Endogone versiformis), and their translation into English. Protologue of E. versiformis (Karsten 1884) and its translation into English. (PDF) [file pone.0023333.s002.pdf]

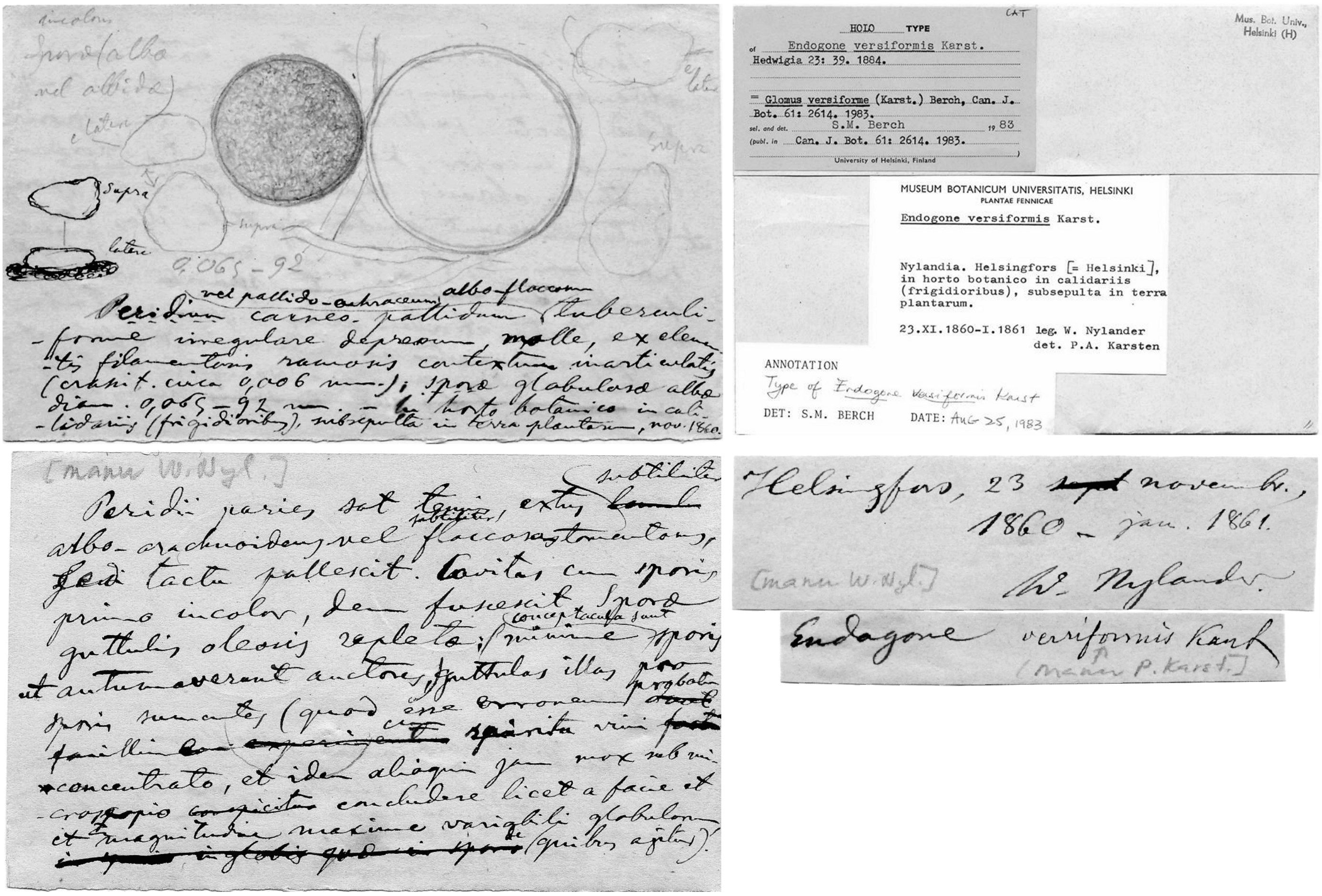

Figure S2: Information accompanying the *Endogone versiformis* type material.

The notes of W. Nylander (23 Nov 1860 – Jan 1861) are transcribed as "*Peridium carneo, pallidum vel pallido-ochraceum, albo-floccosum, tuberculiforme irregulare depressum, molle, ex elementis filamentaris ramosis contextum inarticulatis (crassit. circa 0.006 mm), sporae globulosae albae diam. 0.065–92 mm. In horto botanico in calidariis* [as '*calilidariis*'] *(frigidioribus), subsepulta in terra plantarum, nov. 1860*". This is translated as "Peridium carnose, pale to pale-ochraceous, whitish floccose, tubercular irregularly flattened, soft, composed of filamentous branching aseptate elements (about 0.006 mm [6 µm] thick), white, globular spores with a diameter of 0.065–92 mm [65–92 µm]. In botanical garden in greenhouses (temperate), buried in the soil of plants, Nov. 1860."

Continued on the reverse with notes that are very difficult to transcribe. We transcribe the Latin as: "*Peridii paries sat tenuis, extus subtiliter albo-arachnoideus vel floccoso-tomentosus, sed tactu pallescit. Cavitas cum sporis primo incolor, dein fuscescit. Sporae guttulis oleosis repletae; conceptacula sunt (minime sporis ut autunaverant auctores,) guttulas illas pro sporis sumentes (quod esse erroneum probatum facillime cum spiritu vini concentrato, et idem alioquin iam mox sub microscopico concludere licet a facie et* [as 'et et'] *magnitudine maxime variabili globulorum (quibus agitur)!*" The following translation is based on this transcript, whereas it should be kept in mind that many words were difficult to read or were misspelled: "Wall of the peridium moderately thin, outer layer finer, white-interwoven or floccose-tomentose, but when touched becoming yellowish. Interior containing the spores initially colorless, then darkening. Spores filled with oily guttules; being within a receptacle these guttules (small spores by earlier authors) appearing to be spores (which is an artefact easily tested with concentrated ethanol, and the same in general immediately then can be seen under the microscope concluding from appearance and greatly variable size of the globules (which lead to that interpretation)."

The protologue of *Endogone versiformis* [Karsten PA (1884) Fragmentia mycologica XII. Hedwigia 23: 39–40], written as "*Peridia tuberculiformia, irregularia, mollia, ex hyphis ramosis inarticulatis, circa 6 mmm crassis contexta, carneo-pallida vel pallido-ochracea, albofloccosa, sicca subochraceae, usque ad 1 cm lata. Sporangia subsphaeroidea, albida, diam. 65–95 mmm. Sporae sporoidae. In horto botanico Helsingforsiensi in calidariis (frigidioribus), subsepulta in terra plantarum m., Nov.–Jan.*", is translated as "Peridium tubercular, irregular, soft, composed of aseptate racemose hyphae, about 6 mmm [6 µm] thick, pale flesh coloured or pale ochraceous, white-woolly, dry pale yellowish, up to 1 cm wide. Spores [as 'sporangia'] sub-globose, white, diameter 65–95 mmm [65–95 µm]. Spores spore-like. In the botanical garden Helsinki in glasshouses (temperate), buried in the soil of plants, Nov.–Jan."
