# Supplementary material for: Revealing Natural Relationships among Arbuscular Mycorrhizal Fungi: Culture Line BEG47 Represents Diversispora epigaea, Not Glomus versiforme
Source: PLoS One. 2011 Aug 11;6(8):e23333. doi: 10.1371/journal.pone.0023333 (PMC3154914; doi:10.1371/journal.pone.0023333)
Supplement: Figure S3 — Type collection of Endogone versiformis . Open herbarium packet of the type of E. versiformis, containing dried substrate from potted plants, with spores and fragments of sporocarps and a Petri dish (5 cm diameter) containing sporocarp fragments from the dried substrate. (PDF) [file pone.0023333.s003.pdf]

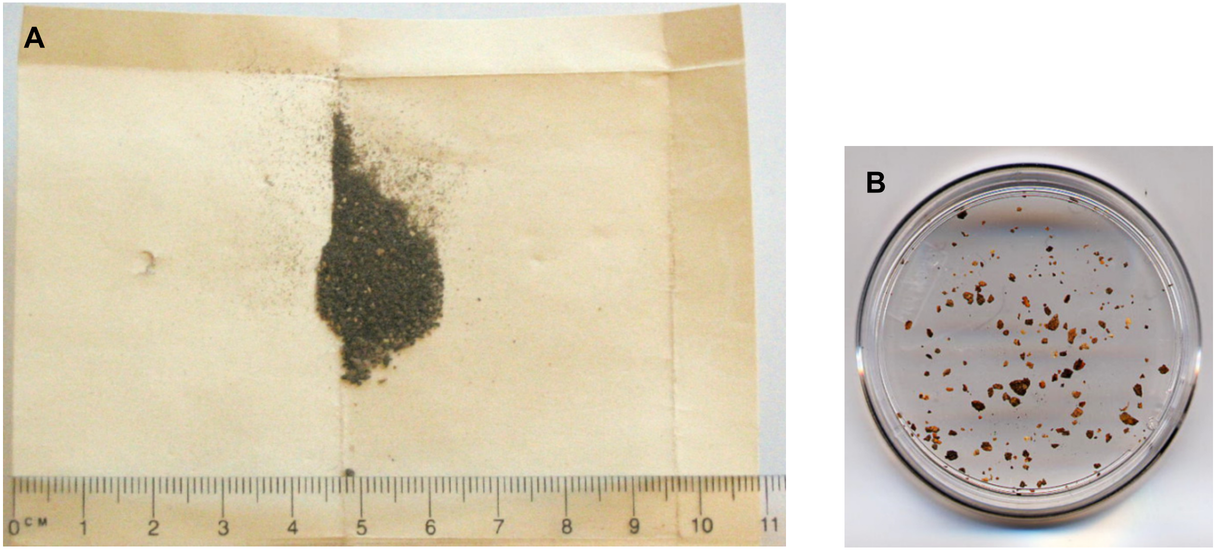

Figure S3: Type collection of *Endogone versiformis*. A. *Endogone versiformis* (= *Glomus versiforme*) type collection. **B.** Sporocarp fragments from the *Endogone versiformis* type collection in a 5 cm Petri dish.
