# Supplementary material for: Revealing Natural Relationships among Arbuscular Mycorrhizal Fungi: Culture Line BEG47 Represents Diversispora epigaea, Not Glomus versiforme
Source: PLoS One. 2011 Aug 11;6(8):e23333. doi: 10.1371/journal.pone.0023333 (PMC3154914; doi:10.1371/journal.pone.0023333)
Supplement: Table S1 — List of studied samples of the Diversispora epigaea ( = Glomus epigaeum ) ex-type culture-line. The culture that was registered as BEG47 is part of the ex-type culture-line of D. epigaea. (PDF) [file pone.0023333.s004.pdf]

Table S1: List of studied samples of the *Diversispora epigaea* (=*Glomus epigaeum*) ex-type culture-line (corresponding to the culture registered as BEG47) samples studied.

Voucher (W)	Culturing Attempt (Att)	Collection Date	Collector	Locality
90	475-1. Pot culture with *Asparagus officinalis* established from a single 'sporocarp' (spore mass) taken from a potted *Araucaria* plant	13 December 1977	B. Daniels	USA, Oregon, Benton Co., USDA- ARS, Horticultural Crops Research Unit (HCRU), Tropical Greenhouse. HOLOTYPE. Trappe 5174. OSC39475
100	475-1. As above	12 April 1979	B. Daniels	As above (HCRU)
407	475-3. No culture data	July 1978 (known only to month)	B. Daniels	As above (HCRU)
1518	475-7. Pot culture with *Sorghum bicolor*	24 January 1981	D. Egel	USA, Ames, Iowa, Iowa State Univeristy Forestry Greenhouse
526	475-6. No culture data except 'Menge 0-1'	13 January 1982	J. Menge	USA, California, University of California, Riverside
1640	475-14. Pot culture with *Allium porrum*	2 December1991	C. Grace	UK, England, Hertfordshire, Harpenden, Rothamsted Experiment Station
1641	475-17. No culture data	1990 (known only to year)	F. Sanders	UK, England, Yorkshire, Leeds, University of Leeds, Dept. of Plant Sciences
1728	475-12. No culture data	19 March 1993	B. Breuinett	Italy, Torino, University of Torino, Departimento di Biologia Vegetale
2336	475-18. Pot culture with *Plantago lanceolata*	11 October 1995	A. Broome	UK, Scotland, Midlothian, Roslin, Forest Research, Northern Research Station
2842	475-20. No culture data	1 October 1996	P. Bonfante	Italy, Torino, University of Torino, Departimento di Biologia Vegetale
3180	475-22. No culture data	15 October 1996	P. Bonfante	Italy, Torino, University of Torino, Departimento di Biologia Vegetale
3206	475-21. Pot culture with *P. lanceolata*	13 January 1999	C. Walker	UK, England, Hampshire, Efford, Horticultural Research International
3221	475-21. Pot culture with *P. lanceolata*	24 February 1999	C. Walker	UK, England, Hampshire, Efford, Horticultural Research International
3537	475-30. Pot culture with *P. lanceolata*	3 November 2000	M. Vestberg	Finland, Vihtavuori, Laukaa Research & Elite Plant Laboratory
3581	475-21. Pot culture with *P. lanceolata*	5 February 2001	C. Walker	UK, England, Hampshire, Efford, Horticultural Research International
3864	475-21. Pot culture with *P. lanceolata*	15 March2002	C. Walker	UK, England, Hampshire, Efford, Horticultural Research International
4475	475-38. No culture data	12 June 2003	B. Blal	France, Dijon, Biorize
4560	475-39. Pot culture with *P. lanceolata*	9 December 2003	C. Walker	Belgium, Louvain-la-Neuve, Catholic University of Louvain
4565	475-40. Pot culture with *P. lanceolata*	15 December 2003	C. Walker	UK, Scotland, Royal Botanic Garden Edinburgh
5164	475-40. Pot culture with *P. lanceolata*	15 April 2006	C. Walker	UK, England, Gloucester (moved from Edinburgh)
5065	475-44. Pot culture with *P. lanceolata*	30 January 2007	G. Bending	UK, England, Wellesbourne, University of Warwick
5066	475-44. Pot culture with *P. lanceolata*	30 January 2007	G. Bending	UK, England, Wellesbourne, University of Warwick
5117	475-45. Pot culture with *Trifolium repens*	08 February 2007	M. Naumann	Italy, Torino, University of Torino, Departimento di Biologia Vegetale
5165	475-45. Pot culture with *T. repens*	28 April 2007	M. Naumann	Italy, Torino, University of Torino, Departimento di Biologia Vegetale
5167	475-45. Pot culture with *T. repens*	28 April 2007	M. Naumann	Italy, Torino, University of Torino, Departimento di Biologia Vegetale

5170	475-46. Pot culture with *P. lanceolata*	16 May 2007	M. Naumann	Italy, Torino, University of Torino, Departimento di Biologia Vegetale
5260	475-45. Pot culture with *T. repens*	1 June 2007	M. Naumann	Italy, Torino, University of Torino, Departimento di Biologia Vegetale
5358	475-45. Pot culture with *T. repens*	25 July 2007	M. Naumann	Italy, Torino, University of Torino, Departimento di Biologia Vegetale
5606	475-55. No culture data	1 February 2009	M. Harrison	USA, New York, Ithaca, Boyce Thompson Institute for Plant Research
5707	475-59. Pot culture with *P. lanceolata*	25 January 2010	C. Walker	UK, England, Wellesbourne, University of Warwick
5708	475-60. Pot culture with *P. lanceolata*	25 January 2010	C. Walker	UK, England, Wellesbourne, University of Warwick
5728	475-56. Pot culture with *P. lanceolata*, *Festuca ovina* agg., *Lotus japonicus* var. gifu	3 March 2010	A. Schüßler	Germany, Martinsried, Ludwig-Maximilians-University Munich
5724	475-48. Pot culture with *P. lanceolata*	9 March 2010	M. Krüger	Germany, Martinsried, Ludwig-Maximilians-University Munich
5725	475-49. Pot culture with *P. lanceolata*	9 March 2010	M. Krüger	Germany, Martinsried, Ludwig-Maximilians-University Munich
5726	475-57. Pot culture with *P. lanceolata*	9 March 2010	M. Krüger	Germany, Martinsried, Ludwig-Maximilians-University Munich
5727	475-47. Pot culture with *P. lanceolata*	9 March 2010	A. Schüßler	Germany, Martinsried, Ludwig-Maximilians-University Munich
5786	475-61. Pot culture with *P. lanceolata*	24 June 2010	C. Walker	UK, England, Gloucester
5835	475-61. Pot culture with *P. lanceolata*	23 September 2010	C. Walker	UK, England, Gloucester
5848	475-66. Pot culture with *P. lanceolata*	26 October 2010	C. Krüger	Germany, Martinsried, Ludwig-Maximilians-University Munich
5849	475-71. Pot culture with *P. lanceolata*	26 October 2010	C. Krüger	Germany, Martinsried, Ludwig-Maximilians-University Munich
