# Supplementary material for: Revealing Natural Relationships among Arbuscular Mycorrhizal Fungi: Culture Line BEG47 Represents Diversispora epigaea, Not Glomus versiforme
Source: PLoS One. 2011 Aug 11;6(8):e23333. doi: 10.1371/journal.pone.0023333 (PMC3154914; doi:10.1371/journal.pone.0023333)
Supplement: Table S2 — Composition of the strict consensus sequences used in the phylogenetic analyses. In strict consensus sequences, site variations are coded by the IUPAC ambiguity code, thus retaining information of the source sequences as degenerate bases, unlike majority rule consensus sequences. (PDF) [file pone.0023333.s005.pdf]

Table S2: Composition of the strict consensus sequences used in the phylogenetic analyses.

Diversispora aurantia	AJ849468 (type culture); FN547655-65, AM713432, EF581860,62,63,80-83 (W4728/Att1296-0 (ex-type soil trap culture)
Diversispora celata	AM713423-25, AY639225-32, EF581865-68 (W4758/Att1292-2, BEG230 = FACE83); AM713426-28, AY639236-41, EF581873-76 (W4757/Att1291-2, BEG232 = FACE272); DQ350448-53 (W5306-07/Att1500-2 = BEG233 = FACE410); AM713417-22, AM713402-04, AY639233-35, AY639306, EF581869-72 (ex-type single spore culture W4718-19/Att1278-2, BEG231 = FACE234)
Diversispora eburnea	AM713405-16,29-31, EF067886-88, EF581877-79 (AZ420A W4729/Att1290-5, ex-type culture)
Diversispora epigaea (all ex-type culture-lineage, originally from Oregon State University, USA, all in the same culture-lineage as BEG47):	AJ132666 (BEG47 from BEG at INRA Dijon, France); AJ276088 (W3221/Att475-21 from C. Walker, Hampshire, England); AM947665, AY842567-69,73,74, FJ461852, FM876814-20, FN547635,36, (W5165/Att475-45 from P. Bonfante, Torino via C. Walker, UK via B. Blal, Dijon, France via INRA, Dijon, France via Rothamsted Experimental Station, UK); FN547666-81 (W3180/Att475-22); X86687, Y17651, FR686938-42 (HC/F-E01 from P. Bonfante, Torino, Italy via J. Trappe, Corvallis, USA)
***Diversispora* sp. W2423**	AJ301863, AJ276076, Y17644, AJ301860, FR686943-44 (W2423/Att382-16)
***Diversispora* sp. 'NamAri'**	AF185677-81 (in sequence database as *Glomus* sp. AZ237B; in INVAM culture collection as *Glomus intraradices* AZ237B); AF185682,90,91, AF185693-95 (in sequence database as *Glomus* sp. NB101; in INVAM culture collection as *Glomus intraradices* NB101)
***Diversispora* sp. W5257**	FR686945-52, FR686958 (W5257)
Diversispora spurca (all ex-type cultures)	AJ276077 (W3239/Att246-4); AJ276078, Y17649,50, FR686953 (W2396/Att246-4); FN547637-54, FR686954 (W4119/Att246-18)
Diversispora trimurales	FR686956-57 (W4124/Att1152-1)
Redeckera fulva	AM418543-44 (AC/Pohn99-001)
Redeckera megalocarpa (from type material)	AM418551,52 (CL/Guad05-051)
Redeckera pulvinata	AM418549,50 (CL/Mart05-035)
Otospora bareai (thought to be from the type material)	AM400229, AM905318 (assembly of non-overlapping 5' and 3' regions of SSU rRNA gene)
***Acaulospora lacunosa* BEG78**	FR719957, AJ891110-13, AJ510230
***Acaulospora laevis* AU211**	AJ250847, AJ242499, FJ461802
***Acaulospora cavernata* BEG33** (given as *A. scrobiculata* at BEG)	AJ306442, FM876788-91
***Acaulospora* sp. WUM18** (equivalent to INVAM AU103A)	AJ306441, FM876792,93
***Claroideoglomus claroideum* BEG14** (=*Glomus claroideum*)	AJ301851,52, AJ276075, Y17636, AF235007
Rhizophagus irregularis GINCO4695rac-11G2 (AFTOL-ID845) (=*Glomus irregulare*)	DQ322630, AY997054, DQ273828
***Funneliformis mosseae* UT101** (AFTOL-ID139) (=*Glomus mosseae*)	AY635833, AY997053, DQ273793
***Gigaspora rosea* DAOM194757**	X58726, AJ410746,47, FN547571-97
***Pacispora scintillans* W4545** (vouchD1, sample3)	FM876831,32, AJ619952-55
***Racocetra castanea* BEG1** (ex-type culture)	AF038590, AJ002874, AJ313169-75, FJ461867, FN423706,07, U31997,98, Y12076
***Scutellospora heterogama* BEG35**	AJ306434, FM876837-39
***Scutellospora nodosa* BEG4**	FM876833-36, AJ306436
